# Spatial transcriptomic analysis of 4NQO-induced tongue cancer revealed cellular lineage diversity and evolutionary trajectory

**DOI:** 10.3389/fonc.2025.1592044

**Published:** 2025-07-03

**Authors:** Feng Liu, Xiaojun Wu, Shaoqing Yu, Deqiang Cheng, Jun Pan, Xiaodong Wang, Guanzhen Yu, Chaofu Li

**Affiliations:** ^1^ School of Information and Control Engineering, China University of Mining and Technology, Xuzhou, China; ^2^ School of Information Engineering, Yulin University, Shaanxi, Yulin, China; ^3^ Department of Neurosurgery, Fudan University, Shanghai Cancer Center, Shanghai, China; ^4^ Allergy and Cancer Research Center, Tongji Hospital, School of Medicine, Tongji University, Shanghai, China; ^5^ Department of Medical Oncology, Jinling Hospital, Medical School of Nanjing University, Nanjing, China; ^6^ School of Computer Science and Technology, Xidian University, Xi’an, China; ^7^ Medical Artificial Intelligence Laboratory, Zhejiang Institute of Digital Media, Chinese Academy of Science, Shaoxing, China; ^8^ Department of Oncology, Liuzhou Workers’ Hospital, Guangxi, Liuzhou, China

**Keywords:** spatial transcriptomics, 4-nitroquinoline-1-oxide, tongue cancer, artificial intelligence, switch genes

## Abstract

**Introduction:**

Spatial transcriptomic analysis has proposed valuable insights into the behavior of tongue cancer. However, the specific cell types involved in chemically-induced carcinogenesis and the process of tumor development remain elusive.

**Methods:**

We leveraged artificial intelligence (AI) algorithms and spatial transcriptomic sequencing to meticulously characterize the spatial and temporal evolution of 4-nitroquinoline-1-oxide (4NQO)-induced tongue carcinogenesis and intratumor heterogeneity.

**Results:**

An AI classifier effectively categorized dysplastic tongue tissue into 13 distinct groups. Spatial transcriptomics identified 13 corresponding cellular subgroups with unique features within the lesion. Both methods successfully distinguished subtle muscle phenotype and genetic lineage variations induced by 4NQO, despite limited morphological differences. Evolutionary tree analysis revealed the dynamic appearance and disappearance of functionally and genetically diverse cell subgroups during the progression from epithelial dysplasia to in situ carcinoma and invasive cancer. Key findings include the identification of specific switch genes associated with tumor invasion and the revelation of significant intratumor heterogeneity.

**Discussion:**

This spatial transcriptomic analysis of 4NQO-induced tongue cancer provides a detailed characterization of tumor evolution and heterogeneity. It elucidates critical aspects of tongue cancer cell behavior and identifies potential therapeutic targets (switch genes). These findings offer novel insights for improving the diagnosis and treatment of tongue cancer.

## Introduction

Tongue cancer is a critical and potentially fatal condition that necessitates immediate diagnosis and intervention (1). It is essential to raise awareness about the risk factors linked to tongue cancer, such as smoking, alcohol consumption, HPV infection, and unhealthy diet, in order to prevent its development. Early detection of tongue cancer through regular screenings can significantly improve the prognosis and increase the chances of successful treatment. Research into the mechanisms of tongue cancer is crucial for the advancement of targeted treatment options and preventive measures. By understanding the underlying causes of tongue cancer, healthcare professionals can better tailor treatment plans to individual patients and improve outcomes.

One of the main risk factors for human cancer is exposure to carcinogens, including DNA oncogenic chemical carcinogens. Among various chemical carcinogens, 4-nitroquinoline-1-oxide (4NQO) is commonly employed to provoke experimental oral cancer. This water-soluble carcinogen predominantly generates tumors in the oral cavity and esophagus (2). This model accurately represents all phases of oral cancer development and demonstrates comparable histological and molecular alterations as seen in humans. Investigating the possible mechanisms of action of 4NQO in promoting tumorigenesis is vital for comprehending the progression of tongue cancer and creating effective chemopreventive approaches. Various studies have utilized 4NQO animal models for the identification of biomarkers. 4NQO induces significant intracellular oxidative stress and is metabolically activated by cellular enzymes. Various signaling pathways, such as those involved in apoptosis, cell cycle control, and intercellular communication, along with other biomolecules, have shown alterations in 4NQO-induced oral cancer models. While these molecules possess the capability to uncover the mechanisms underlying the development of tongue cancer, there are still many unexplored areas, particularly the lack of spatial information on cell types and tissue structures linked to genetic information.

Spatial transcriptomics is a cutting-edge research technique that offers valuable spatial information on cell types and tissue structures, enabling the discovery of new biomarkers and regulatory genes. In the current study, spatial transcriptomics was utilized on paraffin-embedded samples to analyze the spatial gene expression patterns in 4NQO-induced tongue cancer. The main aim of present study is to identify crucial factors and signaling pathways involved in the progression of tongue cancer, gaining insights into the microenvironment and cell interactions contributing to tongue cancer development. Tongue cancer cells are known to exhibit heterogeneity, with different subgroups of cells potentially influencing treatment responses and prognosis. The second objective was to unveil the heterogeneity of tongue cancer cells, providing a deeper understanding of the intricate process of tongue cancer development. By leveraging spatial transcriptomics, researchers can better comprehend the molecular landscape of tongue cancer and potentially identify novel therapeutic targets for improved treatment outcomes.

## Methods

### Animal models

In a previous study, a 4-Nitroquinoline-1-oxide (4NQO)-induced mouse model of esophageal squamous cell carcinoma (ESCC) was successfully established, demonstrating its efficacy in replicating human cancer development (3). Among the well-established ESCC mouse models, instances of concurrent tongue cancer were observed in some cases. Through careful evaluation, samples with invasive tongue cancer were chosen for inclusion in the current study. This selection process ensures that the study focuses on a specific subset of samples with invasive tongue cancer, allowing for more targeted and precise analysis of this particular aspect of cancer development.

### Patient specimens

This study included 23 treatment-naive East Asian tongue squamous cell carcinoma (TSCC) specimens with paired adjacent histologically normal epithelia (10 matched pairs), comprising a cohort of 15 males (65.2%) and 8 females (34.8%) with median age of 50 years (range 30–76 years). Clinicopathological stratification revealed 5 cases (21.7%) at TNM stage I, 10 cases (43.5%) at stage II, 7 cases (30.4%) at stage III, and 1 case (4.3%) at stage IV, with histological grading showing 14 grade 1 tumors (60.9%) and 9 grade 2 tumors (39.1%). The protocol was approved by the Institutional Review Board of Shanghai Changhai Hospital, Naval Medical University (approval#CHEC2020-095), with written informed consent obtained from all participants.

### Automatic sub-regional segmentation

To automatically identify distinct sub-regions within Whole Slide Images (WSIs), a feature extraction and unsupervised clustering approach was implemented. First, background regions without tissue were filtered out from the selected WSIs using Otsu’s method. The remaining tissue regions were then tiled into 512x512 pixel patches at 20x magnification (0.5 microns/pixel), using a sliding window with a step size of 128 pixels. For feature extraction from these tiles, we utilized UNI (ViT-large) (4), a publicly available, state-of-the-art foundation model pre-trained on a large and diverse dataset of pathology images. Crucially, this model was employed directly as a feature extractor in our study without any further training or fine-tuning. Features were extracted from all generated tiles. To refine the feature set, feature selection was performed based on variance to retain the top 256 most informative features. Subsequently, Principal Component Analysis (PCA) was applied for dimensionality reduction.

Following feature processing, unsupervised clustering was performed on the dimensionality-reduced features using the Leiden community detection algorithm, with the resolution parameter set empirically to 0.1. This entire feature extraction, selection, dimensionality reduction, and clustering pipeline was applied independently to each WSI. The resulting cluster labels for each tile were then mapped back to their original spatial coordinates within the WSI, generating a clustering heatmap. This heatmap visually represents the spatial distribution of the identified clusters, enabling the analysis of spatial patterns and tissue heterogeneity within individual tissue samples without relying on prior annotations or model training on our specific dataset.

### Tissue preparation and quality control

Based on the AI analysis results, representative 4NQO-induced tongue cancer samples and untreated tongue samples were selected for delineating the target areas. After consecutive sectioning, some sections were utilized for quality control (DV200), one section was prepared for high-quality Hematoxylin and Eosin (HE) staining pathology slides to obtain cellular morphological features for subsequent analysis. Adjacent sections were used for 10x Visium CytAssist spatial transcriptomic profiling to obtain spatial *in situ* RNA expression profiles.

### Spatial transcriptomic sequencing

The target areas on the paraffin sections (10μm) were mounted onto 10x Visium Spatial slides and subjected to deparaffinization, HE staining, and image scanning following the recommended experimental protocol by 10x Genomics. Subsequently, the 10x Genomics Visium Spatial Gene Expression Slide & Reagent Kit (PN-1000184) and the Visium Spatial Gene Expression Slide Kit (PN-1000185) were utilized for tissue permeabilization, reverse transcription, cDNA amplification, and DNA library preparation, following the guidelines provided with the kits. The quality-checked DNA libraries were subjected to high-throughput sequencing in PE150 mode.

### Data generation and quality control

The raw data obtained from high-throughput sequencing is formatted as fastq files. To process the Visium spatial transcriptomic sequencing data along with bright-field microscopic images, the official software from 10x Genomics, Space Ranger (version 2.0.1), was employed. This included identifying tissue capture areas on the chip, aligning them to reference genomes (human: GRCh38, mouse), and conducting subsequent analyses. Using the Spatial barcode information, reads from each spot were sorted, and statistical evaluations were performed on metrics such as total spot count, reads per spot, detected genes, and unique molecular identifiers (UMIs) to gauge sample quality. Following the initial quality assessment from Space Ranger, additional quality control and data processing were carried out using the Seurat software package (version 4.1.0). The data underwent normalization via the sctransform function, highlighting features with high variance, and was then organized into an SCT matrix for further analysis.

### Dimensionality reduction and clustering analysis for identifying spatial feature genes

The Seurat package’s FindVariableGenes function was employed to identify the top 3000 highly variable genes (HVGs). These gene expression profiles were subsequently analyzed using Principal Component Analysis (PCA) for dimensionality reduction. To visualize the results, Uniform Manifold Approximation and Projection (UMAP), a nonlinear dimensionality reduction method, was applied. For the identification of marker genes, the FindAllMarkers function in Seurat (test.use = bimod) was utilized. This process involved pinpointing genes that showed increased expression in each cell type relative to other populations, thereby serving as potential markers for those cell types. The identified marker genes were then visualized and analyzed further using VlnPlot and FeaturePlot functions.

### Differential gene and functional enrichment analysis between distinct cell populations

The Seurat package’s FindMarkers function was employed to perform differential gene expression analysis on the selected cell populations of interest. Differentially expressed genes were identified using specific criteria, with a p-value threshold set at less than 0.05 and a fold change greater than 1.5. The genes that met these criteria were subsequently analyzed for enrichment in Gene Ontology (GO) and Kyoto Encyclopedia of Genes and Genomes (KEGG) pathways through hypergeometric distribution testing.

### Spatial quasi-temporal analysis

Different invasive regions within the same tissue section were selected for spatial pseudo-temporal analysis to investigate the invasion trajectory and process of tumor cells. The stLearn (v0.4.11) software was utilized for spatial trajectory inference. Initially, PAGA trajectory analysis based on whole-tissue standardized gene expression data was conducted to uncover relationships within subpopulations. Subsequently, a robust trajectory inference method, diffusion pseudotime (DPT), was employed to calculate pseudotime. The DPT method utilizes a random walk-like approach to measure cell-to-cell transitions. The direction of the trajectory was determined by defining a root node based on the biological process under investigation in the tissue. Finally, the pseudo-space-time distance (PSTD) was computed by integrating gene expression data with physical distances. Using this information, a directed graph was created, and the directed minimum spanning tree algorithm was applied to enhance the graph. This process identified the shortest rooted tree and the connections between nodes within the graph.

### Subtype identification in tongue muscle regions

Experiments were conducted on 12 WSIs from the experimental group and 14 WSI from the control group to identify subtypes in tongue muscle regions. First, The regions of interest (ROIs) in each whole slide image (WSI), specifically the muscle tissue areas, were manually annotated. At 20x magnification, these areas were cropped into 256x256 pixel tiles with a sliding window of 64 pixels. The unsupervised pre-trained pathology image feature extraction model, UNI (ViT-large), was then utilized to extract features from all tiles. Next, a 5-fold cross-validation was conducted, organizing the data by whole slide imaging (WSI). In each iteration, two WSIs from the experimental group and two from the control group were randomly chosen as the test set, while the remaining WSIs served as the training set. Each tile was labeled according to its corresponding WSI. A linear regression model implemented in sklearn version 1.42 was used. For each experiment, the trained model inferred the complete ROIs in the test set, and the subtype of the current ROI was determined by a voting mechanism. Finally, the top 50 features with the highest absolute weights from the fully connected layer of each model were selected, and their intersection was taken, resulting in a set of 32 features. These features were used for t-SNE dimensionality reduction and visualization of randomly selected tiles from all samples to evaluate the algorithm’s effectiveness. Additionally, CAMheatmap visualization was applied to display the model’s prediction results on individual tiles.

### Immunohistochemistry

According to the instructions of each antibody, immunohistochemical staining was performed on paraffin-embedded tongue cancer tissue sections. The tissue sections were scored quantitatively by assessing both the percentage of positive cells and the intensity of staining. The H-score was calculated using the formula: H-score = Σ (Pi x i), where “Pi” denotes the percentage of cells within a given staining intensity category and “i” is the intensity score assigned to that category. These intensity scores typically follow a numerical system, with 0 for no staining, 1 for weak staining, 2 for moderate staining, and 3 for strong staining.

### Statistical analysis

Statistical analyses were conducted using IBM SPSS Statistics (version 16.0) and GraphPad Prism (version 5.0). For comparisons between two independent experimental groups, an unpaired two-tailed Student’s t-test was performed. Quantitative data are expressed as mean ± standard deviation (SD) of three biologically independent replicates. Statistical significance was defined as *p < 0.05 and **p < 0.01.

## Results

### The characteristics of 4NQO-induced tongue cancer tissues

**Figure 1:**
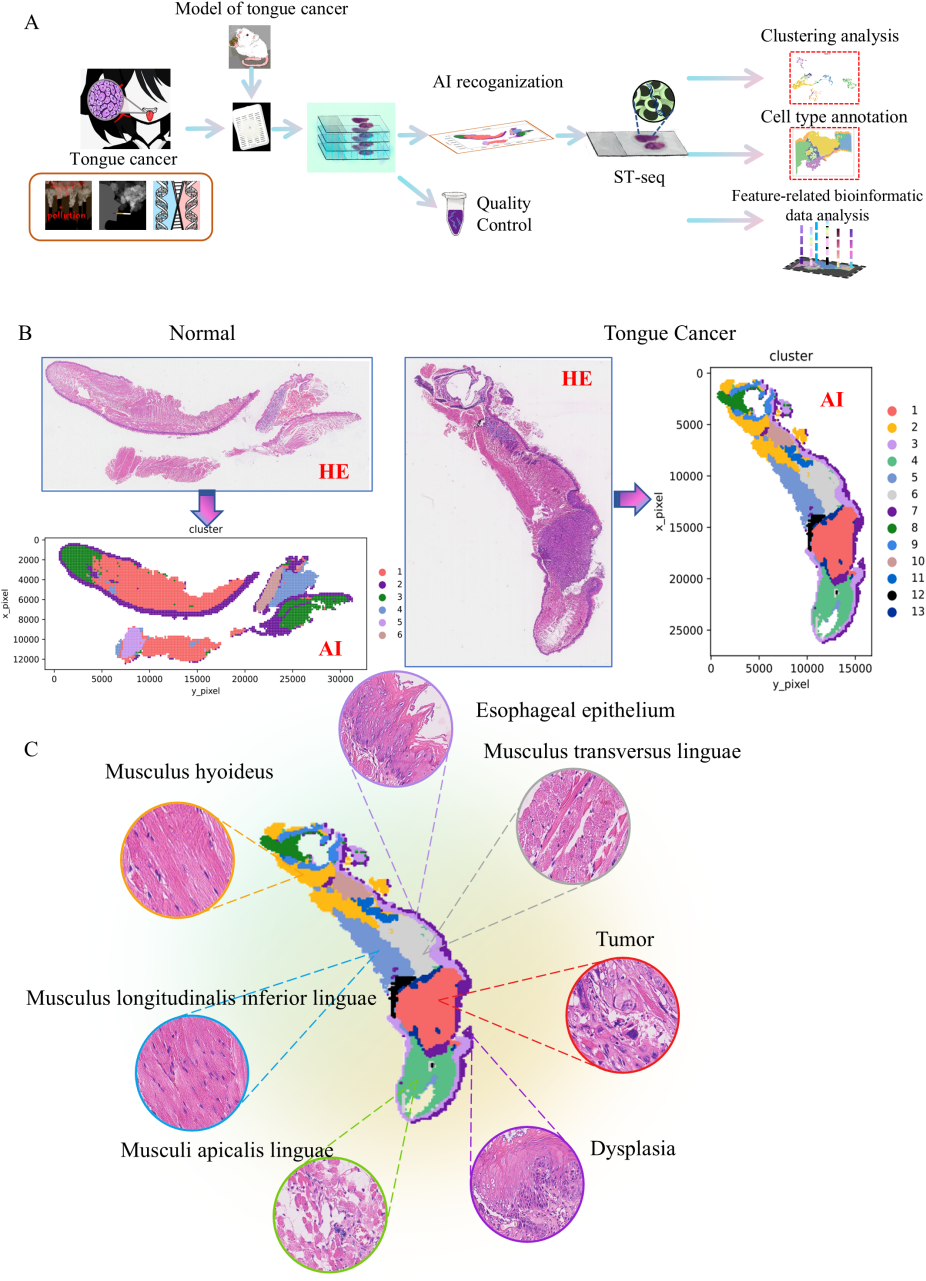
Automatic sub-regional segmentation of 4NQO-induced tongue cancer tissues. **(A)** Schematic representation of the study design. Tumor samples induced by 4NQO treatment and normal tissues were collected and analyzed by an AI algorithm. Representative samples were subjected to spatial transcriptomic analysis. **(B)** The normal tongue was classified into 6 categories, while the tumor tissue was classified into 13 categories by the AI algorithm. **(C)** The most valuable images of these categories from 4NQO-induced tongue cancer tissues.

The study’s workflow is illustrated in **Figure 1A**. To investigate the structural heterogeneity of tongue cancer induced by 4NQO treatment, tumor samples and normal tissues were obtained surgically from the previously established esophageal squamous cell carcinoma (ESCC) mouse model (3). Subsequently, an AI algorithm was developed, which is capable of accurately identifying and distinguishing various structures within tongue cancer and normal samples. Feature extraction and direct clustering were performed on two HE images used for ST-seq analysis. The clustering results successfully segmented the tissue into sub-regions, each displaying distinct morphological characteristics. Feature-related bioinformatic analysis were further analyzed based on the cell type annotation.

As shown in **Figure 1B**, the normal tongue structure exhibits polarity and clear hierarchical organization, which can be classified into 6 categories by the AI algorithm. In contrast, tumor tissue loses its polarity, shows disordered hierarchy, and is classified into 13 categories by the AI algorithm. **Figure 1C** presented the most valuable images of these categories. Under microscopic examination, compared to normal tissue, tongue cancer tissue exhibits 6 characteristics: thickening of the mucosal epithelium, loss of tongue papillae, dysplasia, tumor formation, and ulceration (**Supplementary Figure S1**).

### A spatial transcriptomic atlas of 4NQO-induced tongue cancer and normal tongue tissues

**Figure 2:**
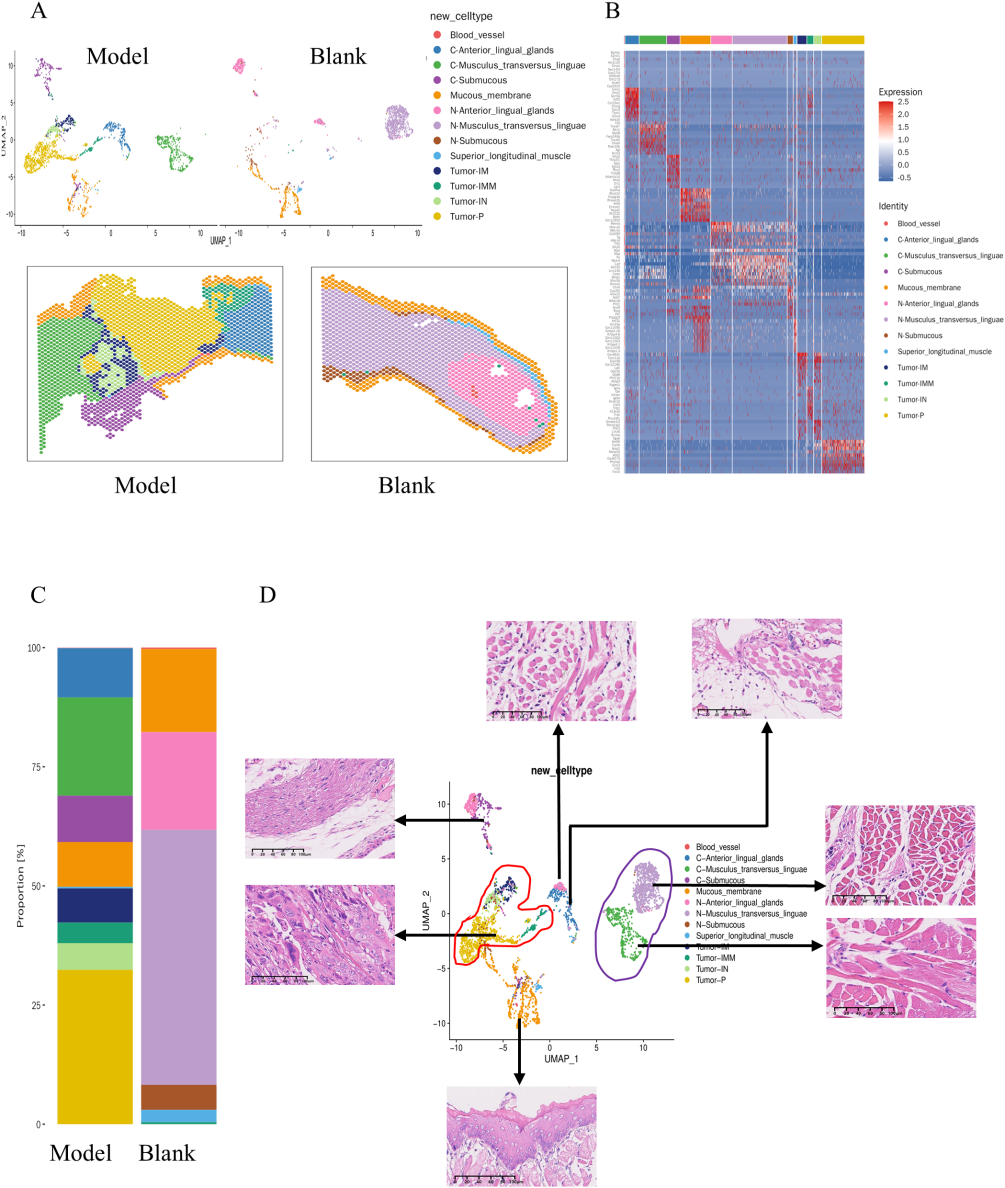
The spatial transcriptomic atlas of 4NQO-induced tongue cancer and normal tongue tissues. **(A)** UMAP plot showing the distribution of cells from 4NQO-induced tongue cancer (Model) and normal tongue tissues(Blank), color-coded by the annotated cell types and pinpoint their specific locations within the tissue. **(B)** Heatmap depicting the expression of specific marker genes across the 13 annotated clusters. **(C)** The distribution of each cluster in the Model and Blank slides. **(D)** The overlapping UMAP plots illustrating the cell distribution in both the Model and Blank slides. Representative Hematoxylin and Eosin (HE) images for selected cluster.

To clarify the cellular composition of tongue cancer induced by 4NQO administration, selected specimens were processed for ST-seq using the 10x Visium CytAssist platform (**Figure 1A**). ST-seq of two samples on two slices was conducted. The number of high-quality spots, average UMI per spot, and average gene number per spot were 2252, 7309, and 2725 for normal tissue, and 2182, 25547, and 6852 for tongue cancer tissue. After filtering ST-seq data to exclude damaged or dead spots, a total of 3741 spot transcriptomes from the two samples were retained for subsequent analysis, generating 19325 genes from normal tongue tissues and 19346 genes from cancer tissue. Following principal component analysis (PCA) for dimensionality reduction and Harmony algorithm to eliminate batch effects, a unified UMAP embedding space was created to visualize graph-based clustering (**Figure 2A**). Cells were categorized into thirteen major cell types based on their respective markers (**Figure 2B**). As depicted in **Figure 2C**, the major cell types differed significantly between tumor and normal tissues, potentially reflecting variances in the stage of tongue cancer initiation. The overlapping UMAPs of normal and tumor tissues highlighted the spatial distribution of cellular composition in a visualized space (**Figure 2D**).

### Spatial transcriptome suggests a carcinogen-type Musculus transversus linguae

**Figure 3:**
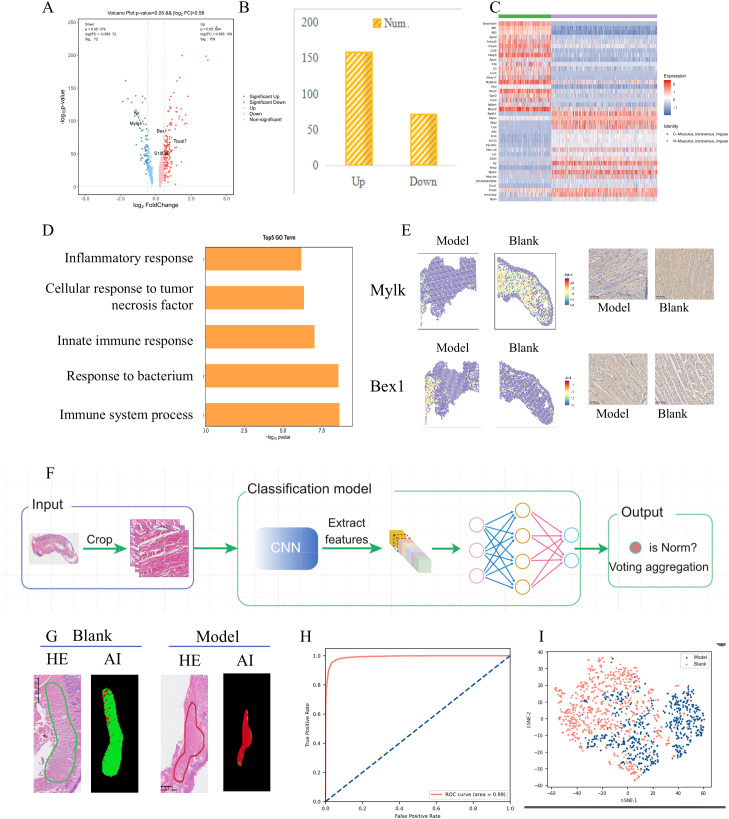
Molecular and morphological differences of the Musculus_transversus_linguae with or without 4NQO administration. **(A)** The volcano plot reveals the gene differences between the Model and Blank samples. **(B)** The number of differentially expressed genes was determined using a p-value threshold of < 0.05 and a fold change greater than 1.5. **(C)** The heatmap displayed the expression levels of selected marker genes within the two clusters. The distribution of each cluster in the Model and Blank slides. **(D)** The top 5 up-regulated Go terms. **(E)** Two selected genes, Myosin light-chain kinase 4 (Mylk4) and BEX1, were validated using immunohistochemistry. **(F)** The workflow illustrating the identification of morphological differences associated with gene expression profiles in then Model and Blank samples. **(G)** Significant differences between the Model and Blank slides disclosed in the two groups. **(H)** An Area Under the Curve (AUC) of 0.990 (95% Confidence Interval: 0.982 to 1.000), with a consistent 100% prediction accuracy at WSI level. **(I)** The distributed Stochastic Neighbor Embedding (t-SNE) was used for dimensionality reduction of the image feature space.

Tumors were visually distinguishable on WSIs, while differences in muscle tissue were not readily apparent. Utilizing UMAP based on spatial transcriptomics highlighted significant variations in gene expression profiles of muscles between normal and tumor-adjacent muscle tissues (**Figure 2D**). The analysis focused on the transversus linguae muscle, with C-Musculus_transversus_linguae representing tissue around the tumor and N-Musculus_transversus_linguae representing normal tissue. Differential genes were identified based on a p-value < 0.05 and FoldChange > 1.5, resulting in a total of 231 differential genes, including 159 upregulated and 72 downregulated genes (**Figures 3A–C**).

Gene Ontology (GO) functional enrichment analysis indicated that the top 5 down-regulated GO terms included regulation of oxidative phosphorylation, electron transport chain, negative regulation of intrinsic apoptotic signaling pathway, keratinization, and proton transmembrane transport. Conversely, the top 5 up-regulated terms included immune system process, response to bacterium, innate immune response, cellular response to tumor necrosis factor, and inflammatory response (**Figure 3D**). Two selected genes, Myosin light-chain kinase 4 (Mylk4) and BEX1, were further validated using immunohistochemistry (**Figure 3E**). Mylk4, a cardiomyocyte cytoskeletal gene, was downregulated in the transverse muscle of the tongue from 4NQO-induced mice compared to normal mice, consistent with previous findings in tissue samples from ischemic cardiomyopathy (5). On the other hand, BEX1, a critical determinant in the cardiac antiviral immune response (6), showed increased expression in 4NQO-affected transverse muscles, indicating impaired inflammatory immune responses in 4NQO-administered mice.

### Subtype identification in tongue muscle regions

The workflow illustrating the identification of morphological differences associated with gene expression profiles is presented in **Figure 3F**. During the subtype identification of tongue muscle regions between the model and blank groups, significant differences between the two groups were observed (**Figure 3G**). The model was validated on all tissue samples from the model and blank groups within the study cohort. Through five-fold cross-validation, it achieved an Area Under the Curve (AUC) of 0.990 (95% Confidence Interval: 0.982 to 1.000), with a consistent 100% prediction accuracy at WSI level (**Figure 3H**). This outcome validates the ability of AI algorithms to distinguish these differences effectively. Furthermore, t-distributed Stochastic Neighbor Embedding (t-SNE) dimensionality reduction of the image feature space revealed a trend of sub-grouping (**Figure 3I**). The Class Activation Map (CAM) heatmap highlighted that differences in muscle gaps were the primary distinguishing feature, providing further confirmation of the reliability of the sequencing results.

### Evolutionary trajectory and switch genes in 4NQO-induced tongue cancer

**Figure 4:**
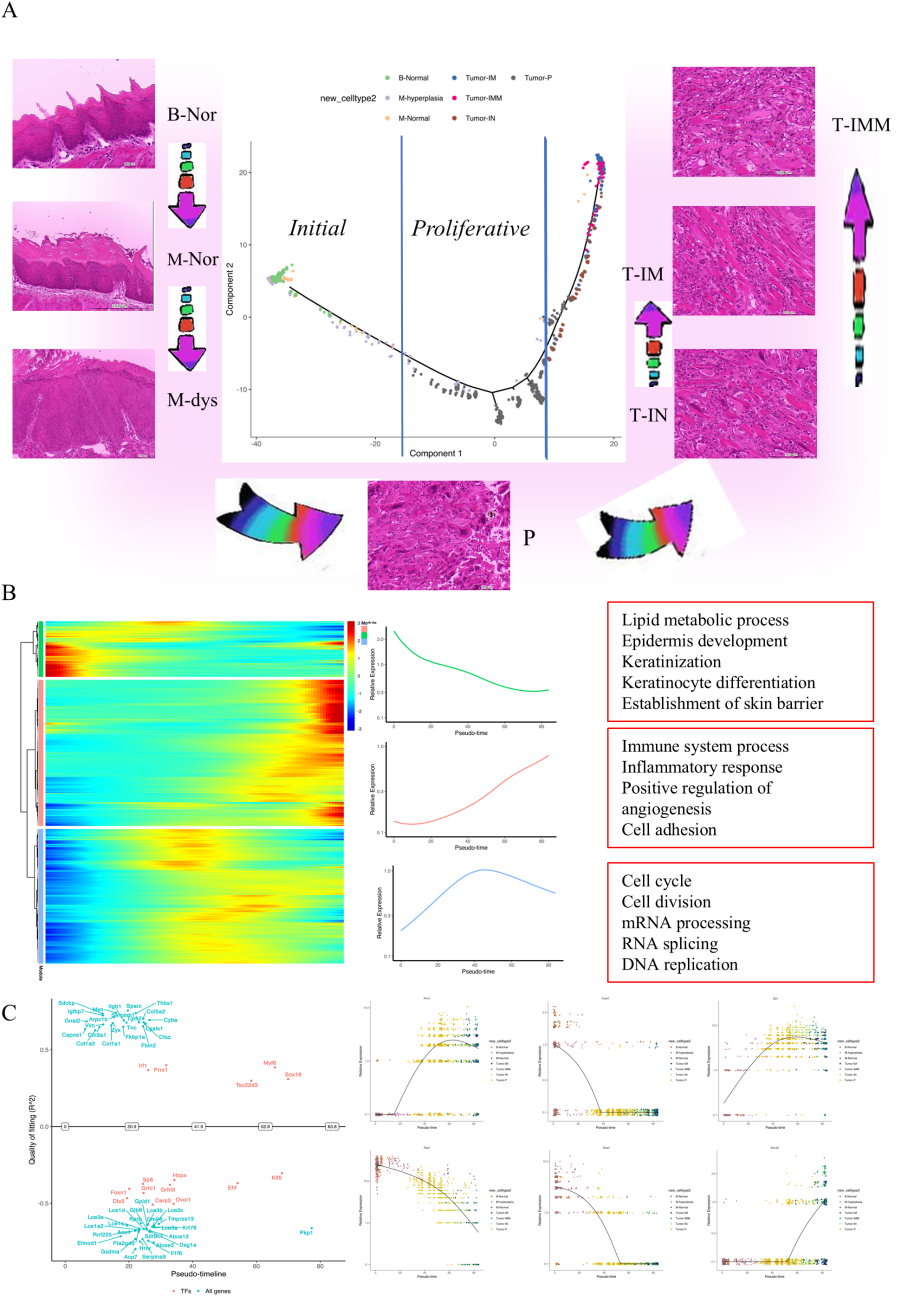
Functional roles and evolutionary trajectories of distinct subclusters. **(A)** Pseudotime-ordered analysis of the ST-seq data depicted identified one major cell fate line, colored by sub-clusters. HE images showing the morphological state of each sub-cluster. **(B)** Heatmap illustrating the dynamic alterations in gene expression over pseudotime (left). There were three kinds of monocles (middle), with the top 5 functions displayed in each monocle(right). **(C)** The dynamic expression of selected genes over pseudotime, represented by color-coded sub-clusters. B-Nor, Blank-normal epithelium; M-Nor, Model-normal epithelium; M-dys, Model-dysplasia; P, Model-primary tumor; T-IM, Tumor-invaded in the muscle on the left; T-IN, Tumor-invaded into the muscle on the left; T-IMM, Tumor-invaded in the muscle on the right.

Based on gene expression patterns across spatial locations within the tissue, The pseudo-time trajectory of 4NQO-induced tumorigenesis and the development of tongue cancer was illustrated (**Figure 4A**). Integrating cellular morphology, the initial stage was characterized by three subclusters representing tongue epithelium from normal tongue, tongue epithelium from 4NQO-damaged tongue, and epithelial dysplasia, which transitioned into the next phase. The second phase denoted the proliferative stage, dominated by a subcluster representing the primary tumor. The third stage represented the invasive phase, where tumor cells invaded into the muscle (IM, left; IMM, right) and within the muscle (IN, left). The heatmap of pseudo-temporal gene expression revealed three modules of key genes involved in the pseudo-time trajectory (**Figure 4B**). Genes in cluster 1, associated with lipid metabolic processes, epidermis development, keratinization, keratinocyte differentiation, and establishment of the skin barrier, exhibited decreased expression with the initiation of tongue cancer. This suggests a gradual disruption of the normal structure and function of the tongue following 4NQO administration. Cluster 2, characterized by increased gene expression, included genes related to the immune system process, inflammatory response, angiogenesis regulation, cell adhesion, and chemotaxis, indicating enhanced tumor invasion capabilities. Cluster 3 encompassed genes involved in cell cycle, cell division, mRNA processing, RNA splicing, and DNA replication. The expression of genes in cluster 3 increased in the early stages of tumorigenesis, followed by a decrease as the tumor progressed. Subsequently, “GeneSwitches” was utilized to identify the temporal switching on or off of genes along the pseudo-time trajectory. Based on the key genes in the pseudo-time trajectory, a gene-switch map was constructed illustrating significant gene switches over time (**Figure 4C**) and the top 10 significant pathways (**Supplementary Figure S2**). Notably, the forkhead transcription factor FOXN1, essential for normal cutaneous and thymic epithelial development (7), and OVOL1, a critical regulator of epithelial lineage determination and differentiation (8), were downregulated upon tumor occurrence. Conversely, SOX18 (9) and Zyx (10), known to enhance migration and invasion abilities in various tumor types, were upregulated as the tumor progressed (**Figure 4C**).

### Evolutionary trajectory within 4NQO-induced tongue cancer

**Figure 5:**
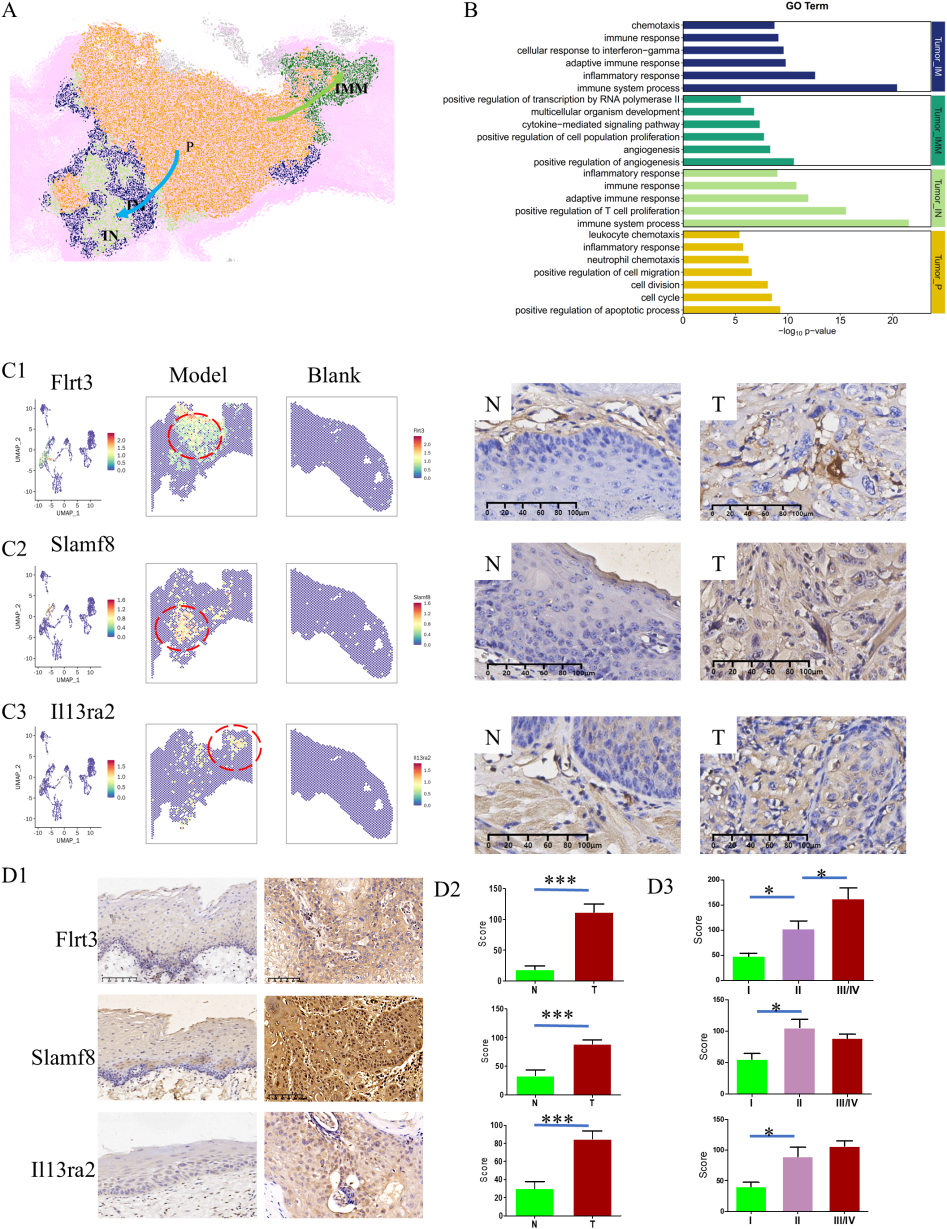
Evolutionary trajectory within 4NQO-induced tongue cancer. **(A)** Spatial distribution of 4 kinds of tumor subclusters. Cluster Tumor-IM/IN was positioned to the left of cluster Tumor-P, while Tumor-IMM was located on the right. **(B)** Gene Ontology (GO) Terms of each subcluster. Clusters Tumor-IM and IN shared similar GO Terms. **(C)** The spatial expression of selected genes(Flrt3, Slamf8, and Il13ra2) in 4NQO-induced tongue cancer and normal tissue based on ST-seq data (left) and validated by immunohistochemistry (right). IHC ×400 **(D)** The expression of Flrt3, Slamf8, and Il13ra2 in human tongue cancer (T) and normal tissues (N) (D1), and the difference between N and T (D2), and the difference across TNM stage (D3). IHC ×200, *P<0.05, ***P<0.001.

Within 4NQO-induced tongue cancer cells, four distinct clusters were identified. The cell trajectory depicted in **Figure 4A** revealed that cluster Tumor-IM and Tumor-IMM both originated from cluster Tumor-P, although these two clusters exhibited overlap in the pseudo-temporal trajectory plot. Notably, cluster Tumor-IM was positioned to the left of cluster Tumor-P, while Tumor-IMM was located on the right (**Figure 5A**).In terms of Gene Ontology (GO) Terms, cluster Tumor-P was primarily associated with cell proliferation, while clusters Tumor-IM and IN shared GO Terms related to the immune system process and inflammatory response. The similarities in location and GO Terms suggested a comparable evolutionary trajectory for the tumor cells in these two clusters. However, cluster Tumor-IMM was characterized by GO Terms linked to angiogenesis and cell population proliferation (**Figure 5B**). This was further supported by Kyoto Encyclopedia of Genes and Genomes (KEGG) enrichment analysis, reinforcing the identified findings (**Supplementary Figure S3**).

Immunohistochemistry validation demonstrated higher expression levels of FLRT3 (11) in cluster Tumor-P, SLAMF8 (12) in clusters Tumor-IM/IN, and IL13RA2 (13) in cluster Tumor-IM, aligning with the aforementioned results (**Figure 5C**). Critically, validation in human TSCC specimens confirmed tumor-specific overexpression of all three biomarkers compared to adjacent normal tissues(P<0.001), with expression levels progressively increasing across TNM stages (p<0.05), most prominently observed for FLRT3 (**Figure 5D**). These findings offer important insights into the possible pathways of tumor invasion, highlighting the relevance of the identified genes in the progression of tongue cancer.

### The gene expression profiles of bizarre cells in 4NQO-induced tongue cancer

**Figure 6:**
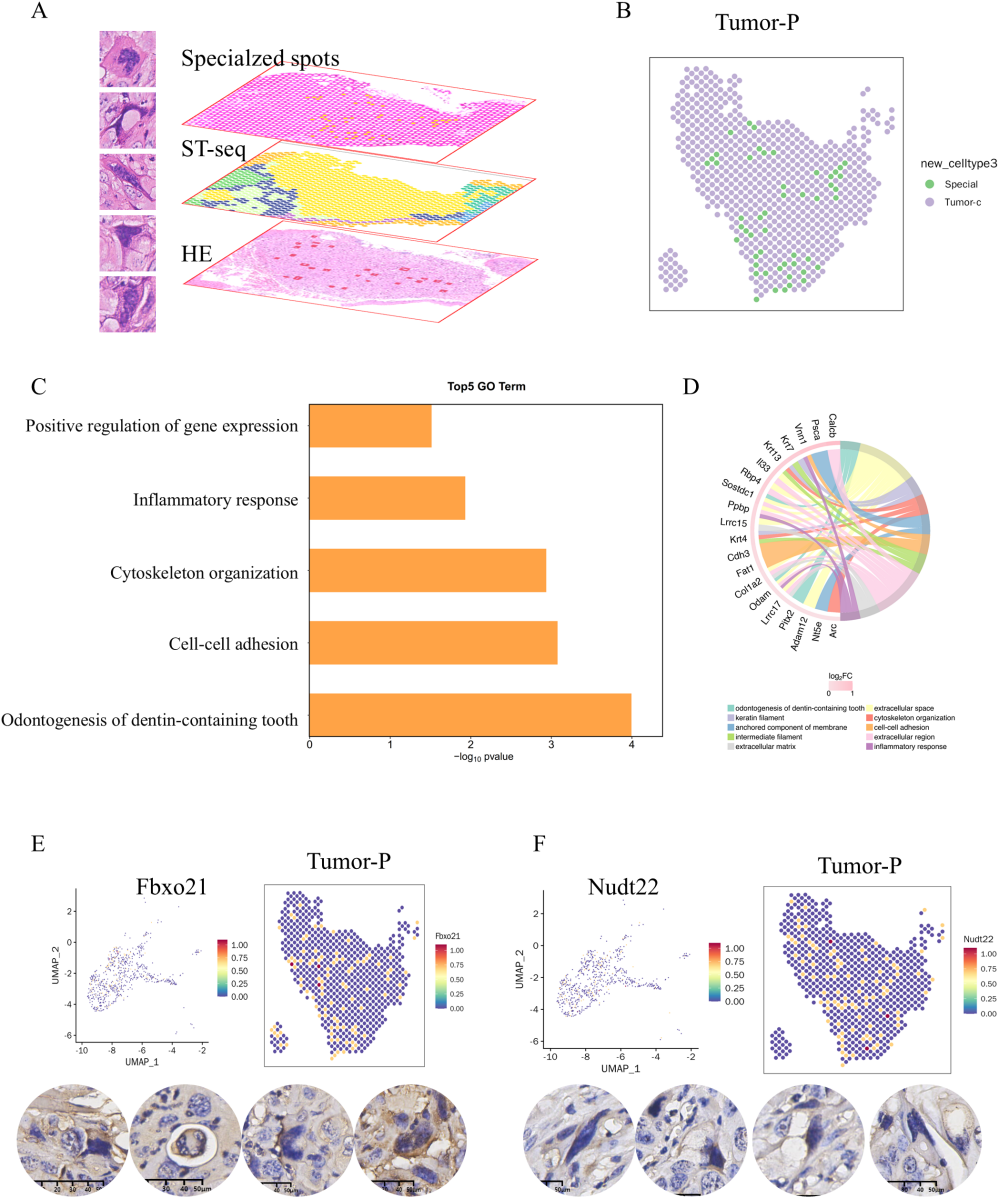
The gene expression profiles of bizarre cells in 4NQO-induced tongue cancer. **(A)** A number of giant cells with abnormal cell morphology were presented in the pathological slides of 4NQO-induced tongue cancer (left) and circled out from the whole spots into a new cluster(right), subjected to further analysis **(B)**. **(C)** Gene Ontology (GO) functional enrichment analysis between the new cluster and cluster Tumor-P. **(D)** The top 20 upregulated genes. **(E, F)** The spatial expression of selected genes(Fbxo21,E; Nudt22, F)in cluster Tumor-P based on ST-seq data (upper) and validated by immunohistochemistry (lower).

One of the most notable discoveries in this study is the presence of giant cells with abnormal cell morphology, characterized by pleomorphic, irregular, enlarged, and deeply stained nuclei in the pathological slides of 4NQO-induced tongue cancer (**Figure 6A**). Given the current limitations in conducting “real” single-cell sequencing using the 10x Visium CytAssist platform, the spots encompassing these cells were delineated, clustered into spot clusters, and compared with those in cluster Tumor-P. Subsequently, differential gene expression analysis was carried out, accompanied by Gene Ontology (GO) functional enrichment analysis (**Figure 6B**). After applying filters of fold change > 1.2 and p-value < 0.05, a total of 117 differentially expressed genes were detected. Among these, 33 genes were upregulated, while 84 genes were downregulated. The top 5 GO Terms associated with these genes were odontogenesis of dentin-containing tooth, cytoskeleton organization, cell-cell adhesion, inflammatory response, and positive regulation of gene expression (**Figure 6C**). These biological functions provide partial insights into the potential reasons and functions of these unusual cells. The top 20 upregulated genes are listed in **Figure 6D**. The protein expression of two genes, Fbxo21 (14)and NUdt22 (15), was validated through immunohistochemistry (IHC) (**Figures 6E, F**).

## Discussion

The 4NQO mouse model is known to replicate the step-wise progression observed in oral squamous cell carcinoma (OSCC) patients, evolving from hyperplasia to dysplasia, papilloma, and eventually invasive SCC. In this study, spatial transcriptomic analysis and artificial intelligence (AI) analysis were conducted to unveil the transcriptome landscape of primary tongue cancer invading the surrounding tissues at both spatial and temporal levels. The research provided a comprehensive overview of the landscape of 4NQO-induced tongue cancer and the adjacent normal tissue. Overall, the tongue cancer samples exhibited significant intra-tumor heterogeneity, along with a highly invasive cellular trajectory. Mechanistically, the progression of tongue cancer necessitated a biological transition of tumor cells from a proliferative state to an invasive state, accompanied by morphological alterations.

The initiation of lesions induced by 4NQO begins in the epithelium of the tongue, mirroring observations in human samples. The development of cancer triggered by 4NQO occurs within its unique microenvironment, akin to human diseases. As a result, the temporal and spatial alterations in the epithelial and stromal compartments provide a robust preclinical platform for investigating the mechanisms underlying the progression and aggressiveness of tongue cancer and for exploring novel therapeutic approaches. Traditionally, histological analysis of H&E stained paraffin sections of tongue tissues is conducted by a trained pathologist using a microscope. The use of a digital slide scanner simplifies and enhances objectivity in this process by generating digital images. These images are then processed by AI algorithms to efficiently and precisely identify and quantify specific cell types, histological characteristics, morphological patterns, and biologically significant regions. In this study, an AI algorithm was employed to effectively delineate the spatial distribution of epithelial, tumor, and stromal compartments, and even differentiate the muscle into five subtypes. AI algorithms have demonstrated successful application in genomic analysis using specific datasets to identify cellular patterns, make predictions, and classify genetic variations. Despite the absence of visible abnormalities in the WSIs of 4NQO-induced transverse muscle of the tongue compared to normal transverse muscle, significant differences were revealed through ST sequencing. The AI algorithms successfully distinguished these differences with an accuracy rate of 100%. These findings suggest that exposure to carcinogens, regardless of tumor presence, can lead to lasting systemic damage.

Specific isolation techniques such as laser capture microscopy (LCM) and single-cell RNA sequencing (scRNA-seq) have been utilized to evaluate localized RNA and/or protein levels specific to the epithelium. Spatial transcriptomics (ST-seq) can also target the epithelium, providing insight into the evolutionary trajectory during the development and progression of tongue epithelial cells. This trajectory aligns with the concept that tumor development and progression are complex processes involving multiple genes, steps, and stages. In the initial stages of tumor initiation, genes associated with proliferation and the cell cycle are typically activated (e.g., CDK4, CDK6, Ki67); conversely, in the advanced stages of the tumor, the expression levels of genes related to invasion and metastasis significantly increase (e.g., MMP2, ADAM9, SOX8). Conversely, genes involved in maintaining keratinization and the skin barrier show a continuous decrease (e.g., KRT14, OVOL1). This data suggests that 4NQO-induced carcinogenesis follows an irreversible single path. However, once a tumor forms, the pathways of invasion and metastasis may vary significantly. ST-seq visualization reveals two distinct paths when 4NQO-induced tumors begin to invade: one path associated with the immune and inflammatory response, and the other linked to angiogenesis. This observation suggests that the tumor microenvironment may contribute to the development of invasive squamous cell carcinoma in a new environment, involving the activation of the immune response and the release of angiogenic factors.

The histological changes in epithelial cells induced by exposure to 4NQO impact key cellular processes including proliferation, apoptosis, metabolism, immune cell recruitment, and metastasis.Several genes associated with these processes are evident in the current ST-seq data. In this study, the aim was to further investigate the temporal regulation of gene expression at each stage of progression.A number of critical “switch” genes were identified, including Foxn1, Ovol1, SOX18, among others. The expression of FOXN1, which plays a role in maintaining keratinocyte differentiation, was downregulated in the early stage of 4NQO-induced primary tumors. Another key driver of epithelial differentiation, Ovol1, was downregulated just before the onset of invasion. Interestingly, the downregulation of Ovol1 coincided with the upregulation of SOX18. SOX18, an essential transcription factor, has been found to be increased in various types of human malignant cancers (16). Consistent with the findings showing activation of SOX18 before invasion, studies have shown that overexpression of SOX18 can significantly enhance the invasiveness and migration of cervical carcinoma cells, without affecting proliferative potential. Spatial transcriptomics serves as a powerful tool for gaining insights into the mechanisms of 4NQO-induced carcinogenesis and for identifying critical genes involved in tumor development. However, the functional characterization of these ‘switching genes’ remains largely unexplored. To address this gap, our subsequent investigations will pursue two interrelated objectives: First, we will employ protein interaction network predictions to systematically map potential cooperative mechanisms between the identified transcriptional regulators and clinically validated therapeutic targets. Second, leveraging longitudinal 4NQO carcinogenesis models in ongoing orthogonal studies, we will prioritize these dynamic regulatory nodes for functional validation. This dual approach will specifically focus on elucidating the temporal coordination of molecular switches during malignant progression.

However, there are several limitations to this study. Firstly, the tissue samples utilized were relatively small in number, and while the data obtained is extensive, it may contain mixed signals and not be universally applicable. Increasing the sample size is crucial to address this issue. Secondly, all samples originated from mouse tongue tissue induced by carcinogens. Although the 4NQO-induced mouse model of oral squamous cell carcinoma (OSCC) mirrors human OSCC, large-scale clinical validation (PDX, PDC model, etc.) is necessary if these models are to be used as targets for tumors or prognostic markers. Additionally, while important switching genes in tumorigenesis and progression have been identified, further investigations are needed to elucidate the underlying molecular mechanisms. While the current spatial transcriptomic analysis in this study is based on population-level data from tissue sections, future methodological advancements should prioritize the adoption of single-cell spatial transcriptomics (scST) (17). This cutting-edge approach holds promise for precisely delineating cellular subpopulation dynamics and their microenvironmental regulatory networks. Furthermore, as emerging technologies progressively overcome current technical constraints in single-cell spatial data acquisition, we anticipate achieving three-dimensional multimodal integration that seamlessly combines: (1) high-resolution morphological features, (2) comprehensive genomic profiling, and (3) spatial context preservation. The ongoing development of next-generation spatial transcriptomic platforms is expected to enable true single-cell resolution sequencing, thereby establishing a unified analytical framework that bridges histological architecture with molecular signatures.”. Although HPV status analysis was not included in this study for the time being, the existing evidence indicates that HPV infection (especially HPV16) is an important cause of head and neck tumors, accounting for 30.8% of global oropharyngeal cancer cases (IARC data) (18, 19). Hpv-positive patients presented unique molecular characteristics (such as the expression of E6/E7 oncogenic proteins) and a better prognosis (3-year survival rate of 82.4%) 810. Future studies will combine single-cell spatial transcriptome technology (scST) 1 with multimodal detection (HPV DNA+mRNA) to further reveal the heterogeneity of the tumor microenvironment driven by HPV and explore its clinical value as a prognostic marker.

In summary, a spatial transcriptomic analysis of chemical-carcinogen induced mouse tongue cancer was conducted, simulating the progression of human tongue cancer development. Through the integration of morphological observations with sequencing data, the evolutionary roadmap of carcinogen-induced mouse tongue cancer at various developmental stages was mapped, highlighting key expression features. Furthermore, a comprehensive analysis of non-epithelial cells in the tumor microenvironment demonstrated that chemical carcinogens induce extensive irreversible damage to the body. These findings offer insights into the fate changes of different cell types during the onset and progression of tongue cancer, providing valuable clues for understanding the pathogenesis of tongue cancer and identifying potential therapeutic or prognostic targets.

## Data Availability

The spatial transcriptomics datasets generated in this study are available in the Genome Sequence Archive (GSA) repository under accession number CRA026708, and can be accessed at: https://ngdc.cncb.ac.cn/gsa/search?searchTerm=CRA026708.

## Supplementary material

Supplementary Figure 1 The characteristics of 4NQO-induced tongue cancer tissues. **(A)** The epithelial tissue of normal tongue. **(B)** Compared to normal epithelium, tongue cancer tissue exhibits 6 characteristics: loss of polarity, thickening of the mucosal epithelium(b1), loss of tongue papillae(b2), dysplasia(b3), tumor formation(b4), and ulceration(b5).

Supplementary Figure 2 The gene-switch map illustrating the top 10 significant pathways over time.

Supplementary Figure 3 Kyoto Encyclopedia of Genes and Genomes (KEGG) enrichment analysis of each subcluster.
