# Supplementary figures and images for: Spatial transcriptomic analysis of 4NQO-induced tongue cancer revealed cellular lineage diversity and evolutionary trajectory
Source: Front Oncol. 2025 Jul 3;15:1592044. doi: 10.3389/fonc.2025.1592044 (PMC12267228; doi:10.3389/fonc.2025.1592044)

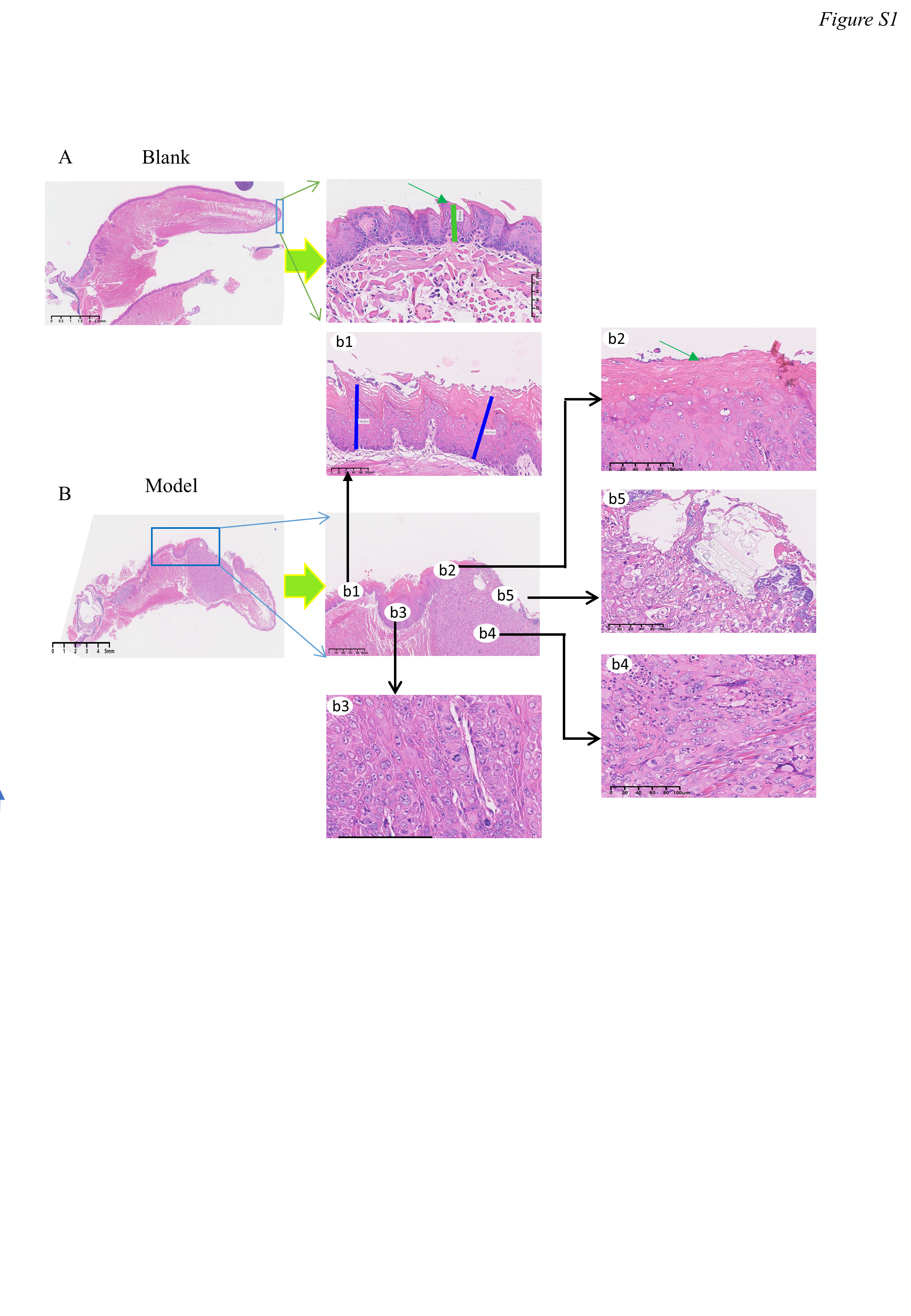

Supplement: Supplementary Figure 1 — The characteristics of 4NQO-induced tongue cancer tissues. (A) The epithelial tissue of normal tongue. (B) Compared to normal epithelium, tongue cancer tissue exhibits 6 characteristics: loss of polarity, thickening of the mucosal epithelium(b1), loss of tongue papillae(b2), dysplasia(b3), tumor formation(b4), and ulceration(b5). [file Image1.tif]

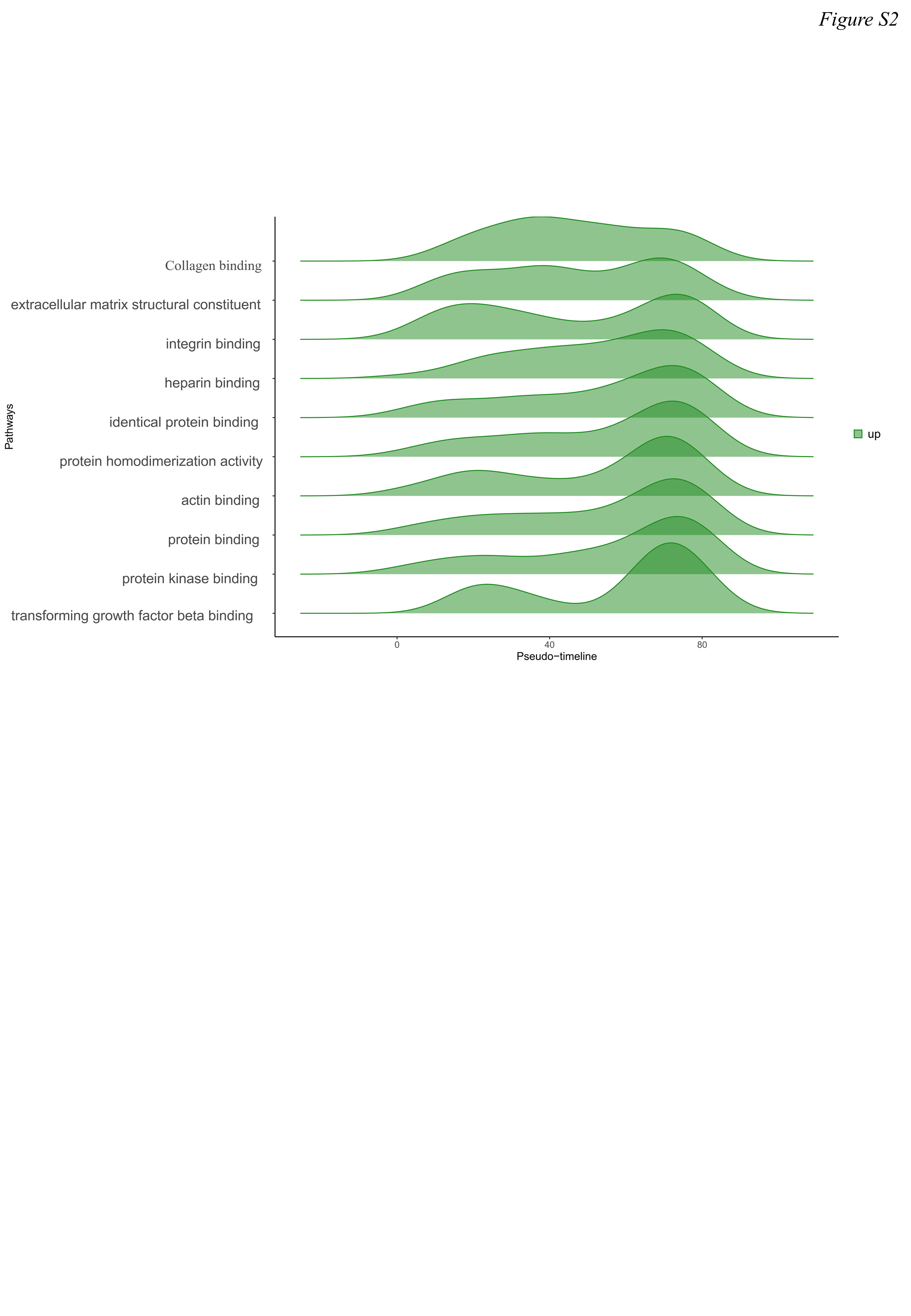

Supplement: Supplementary Figure 2 — The gene-switch map illustrating the top 10 significant pathways over time. [file Image2.tif]

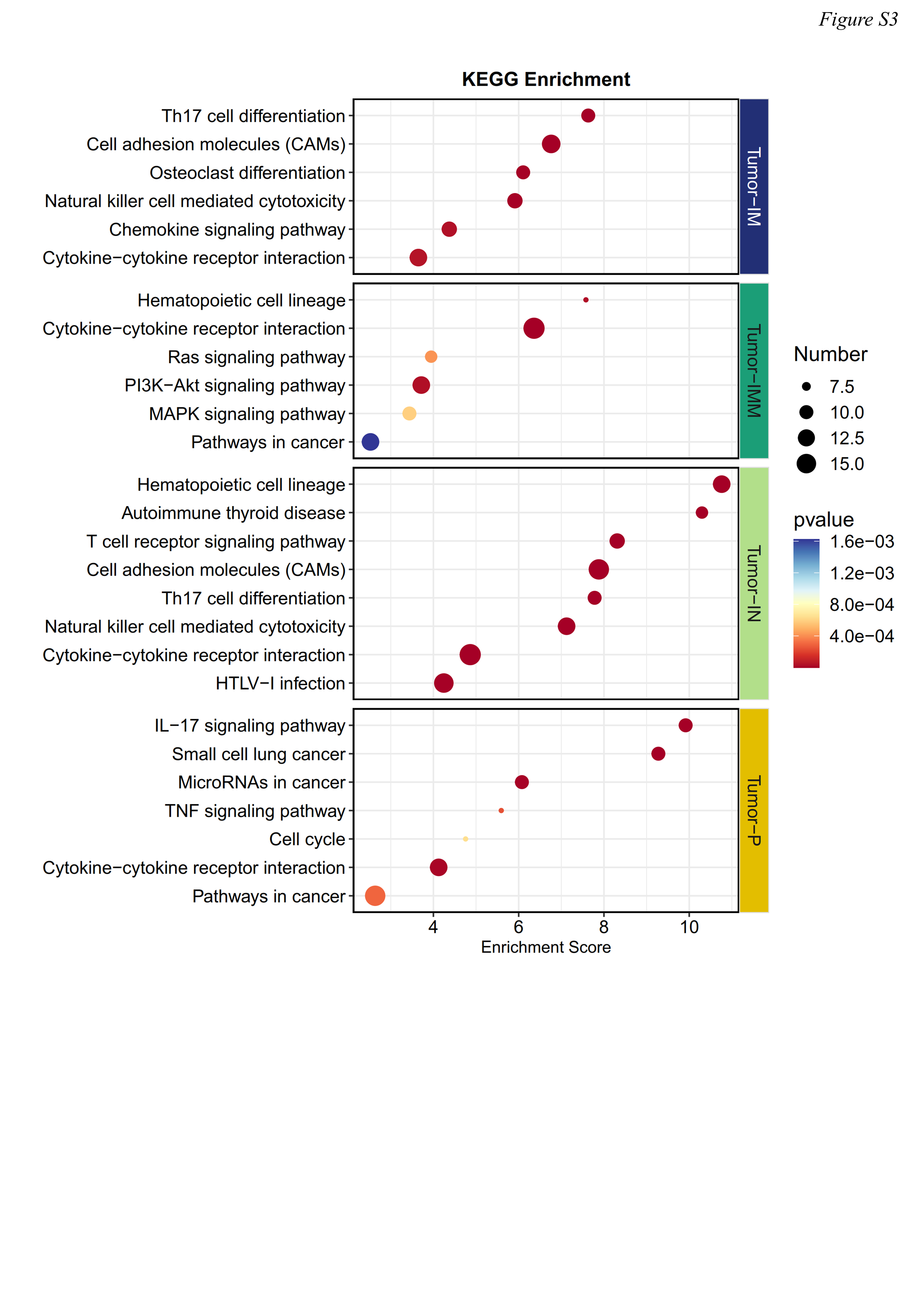

Supplement: Supplementary Figure 3 — Kyoto Encyclopedia of Genes and Genomes (KEGG) enrichment analysis of each subcluster. [file Image3.tif]
